# Platelet reactivity after clopidogrel loading in patients with acute ischemic stroke

**DOI:** 10.3389/fneur.2022.887243

**Published:** 2022-08-24

**Authors:** Yukiko Enomoto, Kenji Shoda, Daisuke Mizutani, Hirofumi Matsubara, Yusuke Egashira, Toru Iwama

**Affiliations:** Department of Neurosurgery, Gifu University Graduate School of Medicine, Gifu, Japan

**Keywords:** antiplatelet, platelet function testing, stroke, CYP2C19, clopidogrel

## Abstract

**Objective:**

It remains unclear when sufficient antiplatelet effect is achieved after administration of a loading dose of clopidogrel in patients with acute ischemic stroke (AIS). This study aimed to evaluate the clopidogrel response in patients with AIS identified by the platelet function test (PFT).

**Methods:**

P2Y_12_ reaction unit (PRU) values measured using VerifyNow at baseline and at 6, 24, and 72 h after administration of a loading dose (300 mg) of clopidogrel were compared between patients with AIS and those of other cerebrovascular diseases (CVD). The prevalence of clopidogrel abnormal response and its associated factors were examined.

**Results:**

The PRU value was significantly reduced with time in the other CVD group (*p* < 0.0001), and also in the AIS group (*p* = 0.0073). The PRU values were significantly higher in the AIS group than in the other CVD group (*p* < 0.0001 between the groups, baseline: 314 ± 53 vs. 284 ± 62, *p* = 0.35; 6 h: 290 ± 66 vs. 214 ± 71, *p* = 0.016; 24 h: 270 ± 75 vs. 190 ± 70, *p* < 0.0001; and 72 h: 231 ± 76 vs. 163 ± 93, *p* = 0.105). The prevalence of clopidogrel hypo-responder (PRU > 240 at 24 h after administration) was higher in the AIS group (79 vs. 33%, *p* < 0.0001) and associated with the baseline PRU value but not with the cytochrome P450 2C19 genotype or clinical ischemic events.

**Conclusions:**

Residual platelet reactivity at 24 h after clopidogrel loading was substantially higher in patients with AIS than in patients with other CVD. In addition, most patients with AIS were judged to be hypo-responders on PFT. This should be carefully interpreted in patients with AIS because of poor specificity for predicting clinical ischemic events.

## Introduction

Antiplatelet treatment with clopidogrel in combination with other antiplatelet agents such as aspirin is widely used for preventing recurrent ischemic stroke. Two major randomized control trials to evaluate an early initiation of combination antiplatelet therapy with clopidogrel and aspirin, as compared with aspirin alone, for patients with minor ischemic stroke (NIHSS score of ≤ 3) or transient ischemic attack have shown a reduction in the risk of recurrent stroke (1, 2). Patients assigned to the combination antiplatelet therapy group received a 300- or 600-mg loading dose of clopidogrel within 24 h of onset because clopidogrel is a pro-drug whose activity depends on cytochrome P450 (CYP) 2C19 metabolism. The early initiation of clopidogrel at loading dose is one important key to preventing recurrent ischemic stroke in the minor ischemic stroke population, but it is not clear for the major ischemic stroke population such as patients with acute ischemic stroke (AIS) with large-vessel occlusion (LVO). Mechanical thrombectomy using a stent retriever, the standard of care in patients with LVO-AIS (3), is an effective technique for the patients with cardioembolic etiology which is most of the LVO-AIS etiology, but not always effective in cases of atherosclerotic etiology or arterial dissection (4). They often require rescue stenting and urgent administration of antiplatelet agents, including clopidogrel, which is mandatory to prevent stent thrombosis (5).

Individual responses to clopidogrel vary by CYP2C19 polymorphism (6, 7) or other clinical factors (8–12), and this variability in pharmacodynamic reactivity to clopidogrel may result in adverse events and poor clinical outcomes (13–17); therefore, platelet function tests (PFT) are used to evaluate antiplatelet effects of clopidogrel and provide a chance of switching or changing doses of antiplatelet agents before elective endovascular treatment (13, 14). Systematic reviews and meta analyses have proven the association between platelet reactivity to clopidogrel and clinical adverse events for percutaneous coronary intervention (15–17); however, most studies were *post-hoc* analysis for platelet reactivity assessed at only one point (12–24 h after clopidogrel administration), by which the intervention had been already finished.

The optimum timing of the endovascular intervention requiring stenting for patients with AIS remains unclear because we do not know the duration for clopidogrel to sufficiently inhibit platelet reactivity.

This study aimed to evaluate changes in platelet reactivity after clopidogrel loading to AIS patients and compare it with those in patients with other cerebrovascular diseases (CVD). The secondary aim of this study was to examine factors associated with PFT-identified abnormal clopidogrel reactivity.

## Materials and methods

This study was approved by the relevant Ethics Committee and Institutional Review Board (22-177, 29-217). The institutional review boards approved the exemption in accordance with the Ethical Guidelines for Medical and Health Research Involving Human Subjects in Japan. Written informed consent was obtained from all patients.

### Antithrombotic management

Our protocol of early antithrombotic management for patients with AIS to prevent further progression or recurrence of ischemic stroke depends on the initial diagnosis of stroke etiology. For patients with cardioembolic or embolic stroke from undetermined sources (ESUS), anticoagulant therapy with continuous infusion of unfractionated heparin was the first-line treatment and then switched to oral anticoagulants the next day or after. If close examination later revealed the atherosclerotic cause in those patients, it was switched to antiplatelet therapy. For patients with atherosclerosis etiology defined as large-artery atherosclerosis or small-vessel occlusion (lacuna stroke) on TOAST criteria (18) or patients with arterial dissection, antiplatelet therapy was the first-line treatment. We recommended aspirin or cilostazol for older patients with mild lacuna stroke, and clopidogrel for other patients. Especially for patients who have a potential for neurological deterioration or urgent endovascular treatment (EVT), such as having multiple risk factors, NIHSS score of ≥ 4 points or LVO-AIS), clopidogrel was recommended to start with a loading dose (300 mg). Patients who had already been treated with P2Y12 inhibitors, including clopidogrel, continued to receive the same dose of P2Y12 inhibitors and added other antiplatelet loading doses. The actual medications or dosage was left to the decision of the treating physician.

### Study population

#### AIS group

**Figure 1:**
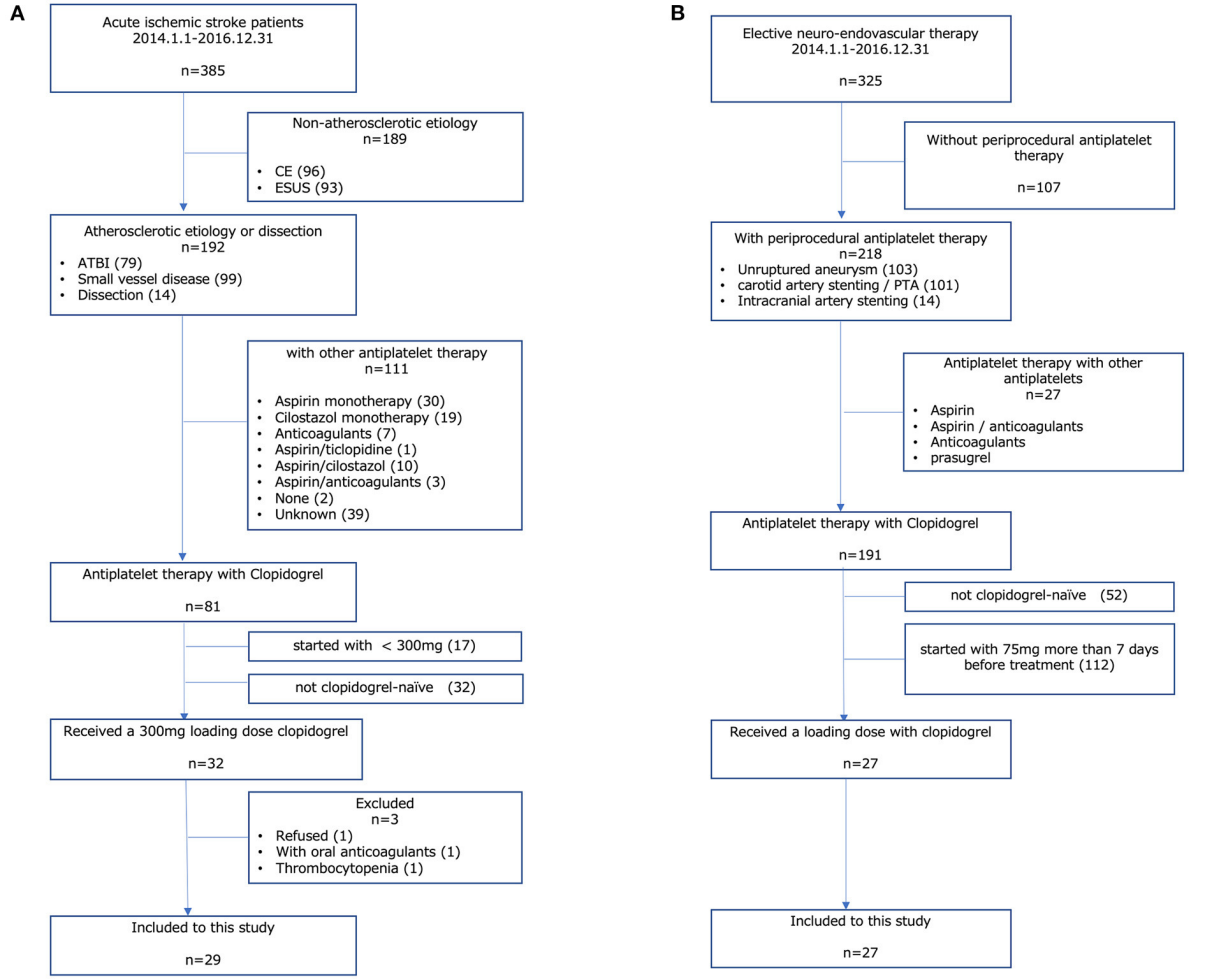
Study population. **(A)** AIS group, **(B)** other CVD group. AIS, acute ischemic stroke; CVD, cerebrovascular disease; CE, cardiac embolism; ESUS, embolic stroke from undetermined sources; ATBI, atherothrombotic brain infarction.

Between January 2014 and December 2016, a total of 385 patients with AIS visited our hospital. Among them, 32 patients received a loading dose (300 mg) of clopidogrel on the day of onset. Patients who received oral anticoagulants (*n* = 1) had thrombocytopenia (*n* = 1) and those who refused to participate in this study (*n* = 1) were excluded from this study; therefore, a total of 29 patients with AIS were included in the analysis (Figure 1A).

#### Other CVD group

Our routine protocol of periprocedural antiplatelet therapy for elective endovascular treatment such as percutaneous transluminal angioplasty and/or stenting for atherosclerotic artery stenosis or stent-assisted coil embolization for cerebral aneurysm is that clopidogrel is started on a maintenance dose for more than 7 days before the procedure. For the cases scheduled for a short period (<6 days) due to the patient's request, clopidogrel is started with a loading dose of 300 mg. In this study period, a total of 325 patients underwent elective endovascular treatment in our hospital. Among them, 27 patients received a loading dose of clopidogrel as periprocedural antiplatelet therapy for elective endovascular treatment and were included in the analysis (Figure 1B).

Written informed consent was obtained from all patients or their families. To confirm the adherence, a nurse checked oral administration or clopidogrel was administered by powder *via* a nasogastric tube for patients with consciousness disturbance or dysphagia.

#### Platelet function test

Clopidogrel reactivity was evaluated by the VerifyNow system (Werfen, Barcelona, Spain), a point-of-care analysis of PFT, which is strongly associated with light transmittance aggregometry (19). Blood samples (5 ml) were obtained at rest from the cubital vein for testing. A citrated tube was filled with the blood, slowly inverted five times, and then inserted into the VerifyNow system.

The value of the P2Y_12_ reaction Unit (PRU), which represents platelets reactivity to clopidogrel, and the Aspirin reaction Unit (ARU), which represents platelets reactivity to aspirin, was measured at 4 points; baseline (before the administration of a loading dose of clopidogrel), and at 6, 24, and 72 h after the administration. The PFT-identified clopidogrel hypo-responder (CHypoR) was defined by a PRU > 240, and hyper-responder (CHyperR) was defined by a PRU <60 at 24 h after clopidogrel administration (13). The reasons for measuring both PRU and ARU was to assess the differences in reactivity and also to confirm the accuracy of the test.

#### CYP2C19 genotype

The CYP2C19 genotype variants were identified with real-time polymerase chain reaction, performed by a commercial entity (SRL, Co. Ltd., Tokyo, Japan). The CYP2C19 gene has several polymorphysims (20), and ^*^2 and ^*^3 alleles are the loss-of-function (LOF) variant (21). The CYP2C19 genotype was classified based on these alleles, as follows: extensive metabolizer (EM, non-carrier of either the ^*^2 or ^*^3 allele), intermediate metabolizer (IM, carrier of either the ^*^2 or ^*^3 allele), poor metabolizer (PM, carrying both the ^*^2 and ^*^3 alleles) (22).

### Data collection and clinical outcomes

Data of variables included patient demographic and clinical characteristics such as age, sex, comorbidities, body mass index, concomitant medication use, and the CYP2C19 genotype were collected from hospital charts.

PRU values at four points and the prevalence of CHypoR and CHyperR were compared between the groups. The associated factors for CHypoR and CHyperR were examined.

Clinical outcomes were measured by clinical and radiological assessments during the patients' hospitalization (12–91 days). Radiological assessment by diffusion-weighted image (DWI) on magnetic resonance images (MRI) was performed 1–7 (median 2) days after administration of clopidogrel for the AIS group or after elective EVT for the other CVD group and when new neurological symptoms developed during hospitalization.

Ischemic event was defined as a development of new ischemic lesions detected by DWI accompanied by any neurological symptom or enlarged primary ischemic area in the AIS group accompanied by an NIHSS score deterioration of ≥4 points. Bleeding event was defined based on the International Society on Thrombosis and Hemostasis's definition of major bleeding during hospitalization. The rates of these events in CHypoR or CHyperR were examined.

### Statistical analysis

Categorical variables are presented as counts and percentages and compared using the chi-square test or Fisher's exact test. Continuous variables were expressed as mean ± standard deviation or median and interquartile range (IQR) and compared with the *Student's*-*t* test. Time-dependent changes to the PRU values within the group were evaluated by a repeated measure ANOVA (the data of PRU values at each timepoint in each group showed normal distribution pattern). The difference of PRU values between the two groups was analyzed using mixed-effect model, followed by a *post-hoc* analysis using the Bonferroni method. Univariate and multivariate logistic regression analyses were used to identify patient characteristics associated with PFT-identified clopidogrel abnormal response; *p*-values of <0.05 were considered statistically significant. All *p*-values were two-tailed. Factors identified as statistically significant in univariate analysis and factors with clinical relevance were included in multivariate analysis. All statistical analyses were conducted using JMP 13.0 (SAS Institute Inc. SAS Institute, Cary, NC).

## Results

Patient characteristics are shown in Table 1. The details of etiology of patients in the AIS group were as follows; large-artery-atherosclerosis (*n* = 23), small-vessel occlusion (*n* = 3), and arterial dissection (*n* = 3). Urgent EVT was performed in 13 patients. The median NIHSS in the AIS group was 5 (IQR 4–8) points, and the median time from onset was 8 (IQR 5.5–10) h. The details in the other CVD group were cerebral aneurysm (*n* = 10), intra-/extra-cranial artery stenosis or dissection (*n* = 17).

**Table 1:** Patients' demographic and clinical characteristics.

**Variable**	**Total** **(*n* = 56)**	**AIS** **(*n* = 29)**	**Other CVD** **(*n* = 27)**	***p*-value**
Age (mean ± SD)	71 ± 11	73 ± 11	63 ± 14	0.003^*^
Female sex (*n*, %)	26 (46%)	12 (41%)	14 (52%)	0.592
Comorbidities (*n*, %)				
Hypertension	47 (84%)	24 (83%)	23 (85%)	1.0
Diabetes mellitus	15 (27%)	8 (28%)	7 (26%)	1.0
Dyslipidemia	28 (50%)	13 (46%)	15 (56%)	0.593
Smoker (*n*, %)	15 (27%)	4 (14%)	4 (15%)	1.0
Body mass index (mean ± SD)	22.6 ± 3	22.4 ± 3	22.6 ± 3	0.872
CYP2C19 genotype (*n*, %)				
Extensive metabolizer	14 (25%)	5 (17%)	9 (33%)	0.221
Poor metabolizer	11 (19%)	9 (31%)	2 (7.4%)	0.042
Baseline value of verify now (mean ± SD)				
PRU	286 ± 59	314 ± 53	284 ± 62	0.088
ARU	643 ± 82	609 ± 77	590 ± 89	0.444
Other medication				
Aspirin	40 (71%)	26 (89.7%)	14 (52%)	0.003^*^
Cilostazol	17 (30%)	6 (21%)	11 (41%)	0.148
Statin	16 (29%)	9 (31%)	7 (43.8%)	0.771
Clopidogrel abnormal response				
CHypoR	32 (57%)	23 (79%)	9 (33%)	0.001^*^
CHyperR	2 (3.5%)	1 (3.5%)	1 (3.7%)	1
Clinical outcomes				
Ischemic event	5 (8.9%)	4 (14%)	1(3.7%)	0.353
Bleeding event	3 (5.3%)	3 (3.5%)	0 (3.7%)	1

SD, standard deviation; PRU, P2Y12 reaction unit; ARU, aspirin reaction unit; PFT, platelet function test; ChypoR, clopidogrel hypo-responder; CHyperR, Clopidogrel hyper-responder.

* p < 0.05; statistical significance.

AIS group was older and had more combination treatment with aspirin. The percentage of CYP2C19-PM was significantly higher in the AIS group than in the other CVD group (31 vs. 7.4%, *p* = 0.042). The values of PRU and ARU were not different between the group (Table 1).

**Figure 2:**
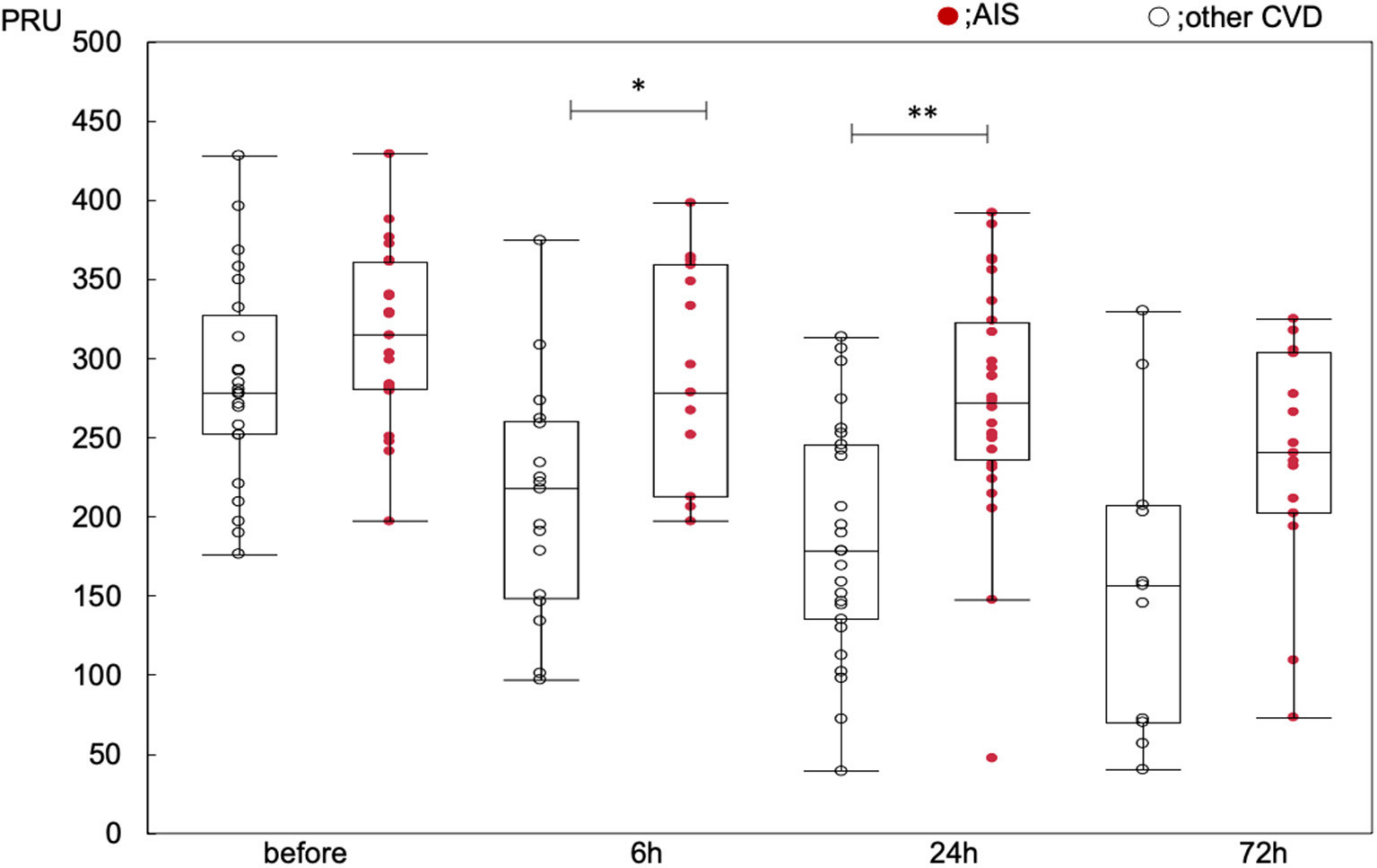
Changes to PRU value over time. *p < 0.05, **p < 0.01, compared within the groups at each timepoint.

Changes to PRU value with time in all patients showed significant reduction after administration of clopidogrel loading dose (baseline: 299 ± 59; 6 h: 249 ± 78; 24 h: 231 ± 83; 72 h: 203 ± 89, *p* < 0.0001). Significant reduction in PRU value with time was observed in the other CVD group (*p* < 0.0001 within the group, baseline-6 h: *p* = 0.013, baseline-24 h: *p* < 0.0001, baseline-72 h: *p* < 0.0001), and also in the AIS group (*p* = 0.007 within the group, baseline-6 h: *p* = 0.678, baseline-24 h: *p* = 0.138, baseline-72 h: *p* = 0.043). The PRU values were significantly higher in the AIS group than in the other CVD group (*p* < 0.0001 between the groups, baseline: 314 ± 53 vs. 284 ± 62, *p* = 0.35; 6 h: 290 ± 66 vs. 214 ± 71, *p* = 0.016; 24 h: 270 ± 75 vs. 190 ± 70, *p* < 0.001; and 72 h: 231 ± 76 vs. 163 ± 93, *p* = 0.105) (Figure 2; Supplementary Tables 1, 2). The PRU values at each time point did not differ between CYP2C19 genotypes (Supplementary Tables 3, 4).

The prevalence of CHypoR was higher in patients with AIS than in patients with other CVDs (79 vs. 33%, *p* < 0.0001). CHyperR was rare (3.5%), observed in only one patient per group.

Ischemic events were observed in five patients with ChypoR. Four of them were large-artery-atherosclerosis patients in the AIS group and the remaining one was a patient with intracranial artery stenosis who underwent PTA in the other CVD group. The patients with ischemic events in the AIS group included 3 females, and their etiology of stroke were artery-to-artery embolism from carotid artery stenosis (*n* = 2), vertebral artery stenosis (*n* = 1) and middle cerebral artery stenosis (*n* = 1). Their CYP2C19 genotype were PM (*n* = 2; ^*^3/^*^3 and ^*^2/^*^2), IM (*n* = 1; ^*^1/^*^2) and EM (*n* = 1; ^*^1/^*^1), and their PRU values at 24 h after the loading dose were 254, 355, 268, 362, respectively.

Remaining one patient with ischemic events in the other CVD group, a female patient in her 70s, was scheduled for an elective percutaneous transluminal angioplasty with stenting for symptomatic intracranial internal carotid artery stenosis. Her PRU value at 24 h after the loading dose was 249, and her CYP2C19 genotype was IM (^*^1/^*^3). EVT was successfully performed 3 days after the loading dose, but she presented motor weakness on her upper limb the day after the procedure. Post-operative DWI revealed a new cerebral infarction.

Bleeding events were observed in three patients in the AIS group. Gastrointestinal bleeding which required urgent endoscopic clip and blood transfusion, was observed in a male patient in his 80s 20 days after onset of stroke due to artery-to-artery embolism from atherosclerotic carotid artery stenosis. His PRU value at 24 h after loading dose was 145, and CYP2C19 genotype was PM (^*^2/^*^2). The other two patients showed subcutaneous hematoma and required blood transfusion at the puncture site of cerebral angiography performed on the day of onset. Their etiology of AIS was large-artery-atherosclerosis, and the two patients immediately received loading doses of clopidogrel (300 mg) and aspirin (200 mg). PRU value at 24 h after loading dose was 48 in a male patient in his 60s with CYP2C19 genotype IM (^*^1/^*^2), and 355 in a male patient in his 80s with CYP2C19 genotype IM (^*^1/^*^2); this patient was the same as the second patient in ischemic events.

### Factors associated with PFT-identified clopidogrel abnormal responder

Multivariate logistic regression analysis of factors associated with CHypoR was performed adjusted for age, CYP2C19 subtype, PRU value before the administration of clopidogrel, group, concomitant use of aspirin, and body mass index. A higher PRU value before the administration of clopidogrel [adjusted odds ratio (aOR) per 1 unit, 1.03; 95% confidence interval (CI), 1.01–1.96, *p* = 0.0010] and the AIS group (aOR, 10.2; 95% CI, 1.50–118.66, *p* = 0.016) were significantly associated with CHypoR (Table 2); however, CYP2C19-PM was not associated (Table 2). Multivariate analysis of factors associated with CHyperR was not performed due to the low prevalence and the absence of significantly associated factors in univariate analysis.

**Table 2:** Factors associated with clopidogrel hypo-response.

**Variables**	**Crude OR**	***p*-value**	**Adjusted OR**	***p-*value**
Age	1.06 (1.01–1.11)	0.020	1.03 (0.97–1.11)	0.373
Female sex	1.89 (0.64–5.56)	0.288		
CYP2C19 poor metabolizer	0.88 (0.23–3.3)	1	0.58 (0.04–9.49)	0.693
PRU value before clopidogrel	1.03 (1.02–1.06)	0.001	1.03 (1.01–1.06)	0.001
AIS group	7.67 (2.31–25.53)	0.001	10.2 (1.50–118.66)	0.016
EVT within 24 h	1.19 (0.34–4.14)	1		
Hypertension	1.84 (0.44–7.76)	0.476		
Diabetes mellitus	1.73 (0.59–5.94)	0.544		
Dyslipidemia	0.79 (0.27–2.31)	0.788		
Aspirin	3.1 (0.93–10.39)	0.078	0.82 (0.08–7.16)	0.857
Cilostazol	0.78 (0.25–2.46)	0.772		
Statin	3 (0.82–10.9)	0.135		
Smoking	0.39 (0.98–1.84)	0.268		
Body mass index	0.84(0.69–1.01)	0.086	0.85 (0.59–1.17)	0.327

Multivariate logistic regression analysis adjusted for factors with a p-value <0.05 in crude analysis or clinical significance in patients.

PRU, P2Y12 reaction unit; AIS, acute ischemic stroke; EVT, endovascular treatment.

## Discussion

Platelet reactivity at 24 h after clopidogrel loading was significantly higher in patients with AIS than in other CVD patients. Although 79% of patients with AIS were judged as CHypoR, it was not associated with clinical events. The PFT-identified platelet reactivity to clopidogrel should be carefully interpreted in AIS patients with AIS.

Among P2Y12 receptor antagonists such as clopidogrel, prasugrel, or ticagrelor which play antiplatelet action by inhibiting the P2Y12 receptor. Clopidogrel is the most widely used for preventing ischemic stroke due to factors such as low cost, insurance coverage, once-daily dosing, and the evidence supporting its efficacy. Clopidogrel, a thienopyridine derivative pro-drug, is inactive in its parent form; it is converted into an active metabolite by the CYP, and irreversibly inhibits the adenosine diphosphate P2Y12 receptor. Clopidogrel metabolism predominantly depends on the CYP2C19 polymorphism, which may influence its speed, affecting the drug's antiplatelet effect. CYP2C19 genotype, the most common factor for clopidogrel hypo-responder (21, 22), was not associated with CHypoR in this study. In East Asian populations, the CYP2C19 polymorphism of the LOF allele (^*^2 or ^*^3), associated with clopidogrel hypo-response, has been reported in >30% of the population, occurring at a rate higher than that observed in other populations (20). These findings support the role of *CYP2C19* genotype in the efficacy of this treatment. Although AIS group contained more PM in this study, CHypoR was much higher than the prevalence of PM, which suggested that non-genetic factors may contribute to clopidogrel hypo-response.

Among patients with minor ischemic stroke or transient ischemic attack, the use of clopidogrel plus aspirin compared with aspirin alone reduced the risk of a new stroke only in the subgroup of patients who were not carriers of the *CYP2C19* LOF alleles in a CYP2C19 genetic analysis of the CHANCE trial (23). For larger artery atherosclerotic stroke patients in subacute phase within 7 days after onset, PRAISE study, a multicenter study in Japan, showed that a higher PRU (>254) was more strongly associated with early recurrence of ischemic stroke than CYP2C19 PM genotype (24). This study suggested that the presence of environmental factors other than CYP2C19 PM genotype influence platelet reactivity, which was similar to the result in our study although the patient backgrounds were different because they included subacute phase stroke patients. In our study, the AIS group and PRU value before the administration of clopidogrel were significantly associated with CHypoR (PRU > 240), but CYP2C19-PM was not associated with CHypoR. Based on these facts, we speculated that systemic activation of platelet aggregation by stress response following acute stroke might affect platelet reactivity.

Campo et al. previously reported that, in patients undergoing a percutaneous coronary intervention, the rate of clopidogrel hypo-response varied over time. Post-clopidogrel PRU values at 1 month independently predicted ischemic and hemorrhagic events, suggesting that the rate of hypo-response might be overestimated in the early phase and that the PRU value may return to its normal range over time (25). The delayed conversion after long-term administration of a maintenance dose has been observed in approximately half of initial clopidogrel normal responders (26). From these reports, PFT may be unreliable for patients in the acute phase even at 24 h after administration; in contrast, it may be suitable for monitoring of long-term treatment. The fact that the prevalence of CHypoR was decreased from 57% (*n* = 32) at 24 h to 35% (*n* = 11) at 72 h in this study supports this speculation.

For elective endovascular treatment, PFT enables clinicians to assess individual platelet reactivity and know the risk of following adverse events. A recent meta-analysis of 12 studies (*N* = 1,464), involving PFT evaluation with VerifyNow prior to flow diverter treatment, revealed that hypo-responders were at a 15% greater risk of thrombotic events and hyper-responders were at a 12% greater risk of hemorrhagic event than normal responders (27). Abnormal platelet reactivity to clopidogrel could be improved by increasing (28) or reducing treatment dose (29), or by adding (30) or switching to other antiplatelet agents (31). Alongside VerifyNow, we have many choices of PFT including light transmission aggregometry (14), TEGs (32), or multiplate (33), but there is no standard method to simulate an *in vivo* platelet response. It remains unclear whether drug adjustments before endovascular treatment based on PFT evaluation can improve patient outcomes (34, 35).

This study had several limitations. First, this was a retrospective analysis of prospectively collected data from a single center. Endovascular procedures and other interventions were performed at the discretion of the attending physician. These differences could affect patient outcomes. Second, the sample size was small. Third, ischemic or bleeding complications were not distinguished from procedure-related complications; it remains unclear whether they were due to antiplatelet or other factor effects. Finally, patient's characteristics in the AIS group were different from the other CVD group in terms of the prevalence of CYP2C19-PM which might affect the results. It may have been necessary to perform a new loading for the AIS group in the chronic phase after the wash-out period to confirm the exact individual response to clopidogrel.

## Conclusions

The PRU value was negatively correlated with time after the administration of a loading dose of clopidogrel; this association was stronger in the other CVD group than in the AIS group. The prevalence of CHypoR was substantially higher in AIS, and associated with baseline PRU value, but not with the CYP2C19 genotype. This definition for CHypoR provided low specificity in predicting clinical events. The present findings suggest that PRU values should be interpreted with caution for patients with AIS.

## Data availability statement

The raw data supporting the conclusions of this article will be made available by the authors, without undue reservation.

## Ethics statement

The studies involving human participants were reviewed and approved by Gifu University Ethics Committee and Institutional Review Board (22-177, 29-217). The patients/participants provided their written informed consent to participate in this study.

## Author contributions

YEn contributed the conception and the design of the work, interpreted the data, drafted the original manuscript, reviewed all suggestions provided by all co-authors (KS, DM, HM, YEg, and TI), approved the final version, agreed to be accountable for all aspects of the work in ensuring that questions related to the accuracy or integrity of any part of the work are appropriately investigated and resolved, composed the final version of the manuscript, and assumed responsibility for final review. TI provided a final overview of the paper and revised a substantial portion of the manuscript. KS, DM, and HM provided a substantial contribution to the acquisition and statistical analysis of the data. YEg provided a substantial contribution to the interpretation of the provided data and contributed with substantial revisions of the original draft. All authors contributed to the article and approved the submitted version.

## Funding

This work was supported by JSPS KAKENHI Grant Number JP25861267.

## Conflict of interest

The authors declare that the research was conducted in the absence of any commercial or financial relationships that could be construed as a potential conflict of interest.

## Publisher's note

All claims expressed in this article are solely those of the authors and do not necessarily represent those of their affiliated organizations, or those of the publisher, the editors and the reviewers. Any product that may be evaluated in this article, or claim that may be made by its manufacturer, is not guaranteed or endorsed by the publisher.
