# Supplementary material for: Platelet reactivity after clopidogrel loading in patients with acute ischemic stroke
Source: Front Neurol. 2022 Aug 24;13:887243. doi: 10.3389/fneur.2022.887243 (PMC9449631; doi:10.3389/fneur.2022.887243)
Supplement: Supplementary file 1 [file Data_Sheet_1.pdf]

Supplementary table

1. The difference of PRU values between the two groups

	degree of freedom (α)	degree of freedom (β)	F (α, β) value	*p* value
timepoint (1)	3	152	13.77	<0.0001
group (2)	1	152	34.28	<0.0001
interaction (1*2)	3	152	1.54	0.206

The data was analyzed using mixed-effect model, and p-values of <.05 were considered statistically significant.

Supplementary table

2. The difference of PRU values at each timepoint between the two groups

time point	difference [95% CI]	*p* value	Bonferroni corrected *p*-value
baseline	29.55 [4.52-63.62]	0.0875	0.35
6 h	76.47 [26.69-126.25]	0.0038*	0.0162*
24 h	84.29 [46.2-122.37]	<0.0001*	<0.0001*
72 h	75.64 [9.74-141.59]	0.0262*	0.1048

These data were obtained by a post hoc analysis using the Bonferroni method, and *p-values of <.05 were considered statistically significant.
