# Supplementary material for: Platelet reactivity after clopidogrel loading in patients with acute ischemic stroke
Source: Front Neurol. 2022 Aug 24;13:887243. doi: 10.3389/fneur.2022.887243 (PMC9449631; doi:10.3389/fneur.2022.887243)
Supplement: Supplementary file 2 [file Data_Sheet_2.pdf]

Supplementary table

3. The difference of PRU values between CYP2C19 genotypes

	degree of freedom（α）	degree of freedom（β）	F（α， β） value	p value
timepoint (1)	3	148	8.96	<0.0001
genotype (2)	2	148	2.84	0.062
interaction (1*2)	6	148	1.39	0.223

The data was analyzed using mixed-effect model, and p-values of <.05 were considered statistically significant.

Supplementary table

4. The difference of PRU values at each timepoint between CYP2C19 genotypes

time point		difference [95% CI]	*p* value	Bonferroni corrected *p*-value
baseline	PM-EM	-26.33 [-79.56-26.89]	0.3242	
	PM-IM	-12.71[-59.39-33.98]	0.5861	
	IM-EM	-13.63 [-55.75-28.49]	0.5178	
6 h	PM-EM	17.27 [-69.38-103.94]	0.6864	
	PM-IM	6.77[-71.3-84.85]	0.8604	
	IM-EM	10.5[-57.28-78.28]	0.7536	
24 h	PM-EM	70.79[5.73-135.85]	0.0335*	0.134
	PM-IM	19.51 [-36.22-75.24]	0.4856	
	IM-EM	51.28[-1.19-103.75]	0.0552	
72 h	PM-EM	102.9[8.06-197.75]	0.0347*	0.1388
	PM-IM	61.11[-18.91-141.03]	0.1274	
	IM-EM	41.79[-42.344-125.93]	0.3148	

These data were obtained by a post hoc analysis using the Bonferroni method, and *p-values of <.05 were considered statistically significant.
